# A Qualitative Approach to a Better Understanding of the Problems Underlying Drug Shortages, as Viewed from Belgian, French and the European Union’s Perspectives

**DOI:** 10.1371/journal.pone.0125691

**Published:** 2015-05-05

**Authors:** Petronille Bogaert, Tomasz Bochenek, Anna Prokop, Andrzej Pilc

**Affiliations:** 1 Department of Drug Management, Faculty of Health Sciences, Jagiellonian University Medical College, Krakow, Poland; 2 Chair of Epidemiology and Preventive Medicine, Faculty of Medicine, Jagiellonian University Medical College, Krakow, Poland; 3 Department of Neurobiology, Institute of Pharmacology, Polish Academy of Sciences, Krakow, Poland; York University, CANADA

## Abstract

The problem of drug shortages has been reported worldwide, gaining prominence in multiple domains and several countries in recent years. The aim of the study was to analyze, characterise and assess this problem in Belgium and France, while also adopting a wider perspective from the European Union. A qualitative methodological approach was employed, including semi-structured interviews with the representatives of respective national health authorities, pharmaceutical companies and wholesalers, as well as hospital and community pharmacists. The research was conducted in early 2014. Four themes, which were identified through the interviews, were addressed in the paper, i.e. a) defining drug shortages, b) their dynamics and perception, c) their determinants, d) the role of the European and national institutions in coping with the problem. Three groups of determinants of drug shortages were identified throughout this study: manufacturing problems, distribution and supply problems, and problems related to economic aspects. Currently, the Member States of the European Union are striving to resolve the problem very much on their own, although a far more focused and dedicated collaboration may well prove instrumental in coping with drug shortages throughout Europe more effectively. To the best of the authors’ knowledge, this is the first qualitative study to investigate the characteristics, key determinants, and the problem drivers of drug shortages, focusing on this particular group of countries, while also adopting the European Union’s perspective.

## Introduction

Drug shortages have been reported for several years, although it is only recently that the reports seem to be gaining prominence in several classes of medicines and in multiple countries [1–4]. The World Health Organization (WHO) described medicine shortages as a complex global challenge, affecting more than 20 countries worldwide [5]. For the purpose of this study, drug shortages were defined as a situation in which the current or projected demand of a medicine at user level is inadequately met. Their origins are complex, diverse and differ among regions and countries [6–8]. A non-exhaustive list of causes for drug shortages was provided recently by Birgli’s research, including those stemming from both unpredictable and predictable reasons [6]. According to this source, the first group of reasons comprises: natural disasters, manufacturing problems, raw material shortages, non-compliance with regulatory standards, packaging shortages, unexpected demand, epidemics, parallel distribution, competitive issues, foreign exchange effects and sovereign issues (financial crisis, debt, default). The second group of reasons comprises: product discontinuation, industry consolidation (mergers and acquisitions), limited manufacturing capacity, just-in-time inventories, rationing/quotas, deliberate shortages used to manipulate prices, market shifts, launches of new competitors, new formulations, or patent expiry.

Regardless of the cause, drug shortages have an impact on all stakeholders in the supply chain. When they occur, patients may not receive their medication, or an alternative treatment will have to be found to substitute for the missing product. This can lead to inconvenience and distress or clinical risks such as medication errors, adverse effects and disease progression. The published empirical evidence is often based on the North American experience, including the USA or Canada, where the effects of medicine shortages have been researched more extensively than in Europe [9–17]. In order to find alternative treatments, pharmacy personnel will have to spend extra time on alternative sourcing for medications [18]. A study carried out in 2011 by Chemist & Druggist indicated that 95% of pharmacists in the UK spent between one and two hours per week tracking out-of-stock medicines [19]. The estimated labour cost linked with management of drug shortages was set at $216 million annually in the USA alone [20]. Furthermore, the cost of purchasing alternative medicines adds to the financial burden of US hospital pharmacies due to reimbursement restrictions [9,10]. It is estimated to cost the US hospitals approximately $215 million annually [21].

The study aimed to analyse, characterise and assess the problem of drug shortages in Belgium and France. A multidimensional character of this issue and the perspective of the European Union (EU) were taken into account. The research tasks focused on investigating present appreciation and interpretation of the drug shortages phenomenon, as well as its determinants and problem drivers.

It is difficult to characterise and assess the problem of drug shortages by using quantitative methods only, due to the following reasons. First of all, there is a scarcity of publicly available data on drug shortages at different levels in the supply chain. Besides, quantitative data is more likely to remain confidential owing to the very controversial nature of the topic. Second, no direct comparison can be made between respective countries, as different definitions of drug shortages are used by the medicine agencies for reporting or recording purposes. Therefore, with a view to exploring and gaining a better understanding of the underlying mechanisms of drug shortages, completing the research gap, as well as assessing organisational and systematic solutions, a qualitative methodological approach was employed in this study.

## Methods

A semi-structured interview was designed to achieve the study objectives. Key professionals within the medicine supply chain or medicine management, e.g. representatives of relevant bodies, organisations, or trade associations, were recruited. The interviewees were grouped relative to their professional roles, and in line with the following criteria:

- national authorities, including medicines agencies, national ministries of health and finance, reimbursement agencies (policy-makers);
- pharmaceutical companies, including original and generic manufacturers;
- pharmaceutical wholesalers, including wholesale distributors, parallel traders, short- and full-line wholesalers;
- hospital and community pharmacists.

During a three-month period in the early 2014, a total of 28 stakeholders in France, Belgium and at the European Union’s (European) level were invited by electronic mail and/or telephone to participate in the study. Purposive sampling of interviewees entailed inviting a minimum of two representatives of each stakeholder group in Belgium, France and at European level. Additionally, the snowball sampling technique was used to recruit the most informative stakeholders. The interviewees who positively replied to the invitation of the research team to participate in the study were asked to name further professionals within the pharmaceutical sector, who have extensive knowledge on the topic, extensive understanding of the problem, as well as those who are or have been professionally involved in developing solutions to the problem.

During the preparatory phase four specialized interview guides were constructed, each of them relevant to the professional profile of a particular group of stakeholders (S1–S4 Text). The interview guides were piloted and subsequently refined. They covered the following topics: understanding the phenomenon of drug shortages, its perceived scope and impact, as well as possible solutions. Additionally, in order to facilitate the process of interviewing, a table depicting the breakdown of reasons for drug shortages was inserted into each interview guide. It was based on the report recently published by Birgli [6]. A table was presented to each study participant during the interview with a request to express their own opinion on the causes of drug shortages and to reflect individually on the typology provided therein. Interviews, lasting ca. 60 minutes each, were audio-recorded and then transcribed verbatim. The online software Transcribe! was used to support the transcription [22]. Transcripts were coded and analysed using MAXQDA software package, Version 11 [23].

The process of analysing the data involved conducting data reduction and applying constant comparison techniques. An open coding approach was used to examine the text and to analyse it by topics. A code was attributed to individual words or sentences, in order to categorize the data according to their meaning and referenced action. The code would often arise directly from the data. Codes emerging from early interviews formed a developing coding taxonomy that was used to analyse subsequent interviews. In some instances, the coding was revised based on the new data derived from the interviews. Re-coding and a discussion took place in an effort to ensure that the codes matched the data accurately. Besides the coding, the process of data reduction entailed writing summaries and memos. Finally, to determine the patterns of response concerning the understanding of the phenomenon of drug shortages by a particular stakeholder group, as well as national and European settings, certain segments of the data were compared within a single case, as well as between various cases.

Several strategies were implemented to ensure scientific rigour in the collection and analysis of the data. A single researcher conducted all the interviews, although regular consultations with other members of the research team were also pursued. Since the interview technique specifically selected for the purpose facilitated a follow-up of the interviewees, should they raise any interest in terms of the actual research questions, while at the same time departing from the set interview guidelines, every interviewer was required to be well versed in the specifics of the area under study.

In order to be able to follow up the problems raised during the interviews, the interviewing author carried out a wide literature review prior to the interviews. The interviewing author recorded additional information, using a reflective researcher’s log and field notes to aid powers of recall, thereby ensuring consistency and accuracy of reporting. Memos were used to keep track of relevant reflections during the analysis of the interviews. These encompassed reflections on theoretical aspects, data analysis and the researcher’s own impressions. All data were synthesized under simple codes, while rigorous theoretical thinking led to further refinement of the explanations, effectively facilitating the attainment of the study objectives.

The limitations typical for qualitative research were duly considered when approaching the issue of external validity or generalizability of the study results [24]. The constant comparison technique was used to ensure quality and internal validity of the study, helping to verify coherence and precision between the codes generated during the analysis and the facts, as well as the problems they referred to. The coding system for all interviews was then revised and optimised. Additionally, some relevant quotations were provided within the study report in order to enhance its overall validity, as well as reflect the actual empirical material with greater accuracy. Besides, such quotations ensured an acceptable level of validity of the study results, in parallel with other measures, such as provision of specific examples. Reliability of analysis was also secured by checking transcripts for any transcription mistakes. Finally, data triangulation was performed to enhance overall credibility of the findings. The phenomenon was studied as understood by interviewees from four different professional perspectives, as well as both national and international settings.

The Jagiellonian University (Krakow, Poland), the University of Sheffield (UK), and the Katholieke Universiteit Leuven (Belgium), granted ethical approval for the study, respectively. Before the interviews were conducted, the interviewees received detailed information on the study design. Written informed consent was obtained from all participants who were assured of data confidentiality and their right to withdraw at any time.

## Results

Overall, 21 semi-structured interviews were conducted in person with the key representatives of various institutions (Table 1). Nine interviewees came from public health authorities. Four interviewees were from the pharmaceutical companies, four represented the wholesale distributors and four represented the pharmacies (or their associations, respectively). Data saturation was achieved with the total number of participants [25]. Qualitative content analysis and data reduction were used to classify the 135 allocated codes into 14 categories and consequently several themes were identified [26]. They comprised: defining drug shortages, their dynamics and perception, their determinants, the role of the European and the respective national institutions in coping with the problem.

**Table 1:** A summary characteristics of the interviewees.

Countries	Representatives* of public health authorities	Representatives of pharmaceutical companies	Representatives of wholesalers	Representatives of pharmacies
Belgium	2	0	1	2
France	6	1	1	1
European Union	1	3	2	1
**Total**	**9**	**4**	**4**	**4**

*Public health institutions include medicines agencies, reimbursement agencies, policy-making institutions.

### Defining drug shortages

The interviewees frequently highlighted overall complexity of the drug shortages phenomenon and the resultant difficulty in agreeing on a common definition. Inconsistency of the nomenclature emerging from the analysis was also acknowledged by the interviewees. One interviewee observed: "How to understand drug shortages is a very complicated issue and subject to some debate, because some regard a shortage as a situation in which one is unable to get hold of a medicine within a reasonable time, whereas others define it as medicine which is not really available on the market at all".

In Belgium, the term “onbeschikbaarheid” (literally translated as unavailability) of medicines is used by national institutions, although other terms, like “tekorten” (shortage) or “stockbreuk” (stockout) are also being used. Most of the stakeholders in France agreed on using the terms “rupture d’approvisionnement” and “rupture de stock” (disruptions of supply and stock, respectively), although their precise meaning was found to be rather confusing, as different definitions were offered when asked for the exact meanings of the terms. A disruption of supply (legislative term) is generally understood as a problem in the distribution chain, whereas a disruption of stock refers to a problem at the manufacturing level. Also other terms—“rupture anticipée”, “rupture constatée” (anticipated and actually observed shortages, respectively)—were used by the French interviewees. An anticipated shortage is reported by a manufacturer, while an actually observed shortage—by a pharmacist. In English, the term shortage is used predominantly by the majority of the interviewees. They highlighted the importance of using a common and well-understood terminology. As an industry representative stated: “We feel it is the basic block upon which you are going to build. If you do not agree on this, you will face problems with the interpretation and quantification later on."

A great diversity of opinions regarding the actual level at which drug shortages should be assessed was found to be the second challenge. In Belgium, in line with applicable legislation, a shortage is deemed to occur when a company at issue is unable to respond to any delivery requests. Under French law, a shortage is construed as the inability of a pharmacy to dispense a medicine to a patient. However, several interviewees noted that a pharmacist might not be able to dispense a particular medicine only because his preferred wholesaler happens to be unable to deliver the product, although it may well be available at a pharmacy nearby. At the European level, several interviewees agreed on a patient-centred approach, believing a drug shortage is "a disruption in supply of medicines, affecting the patient’s ability to access the required treatment within the appointed time-frame."

The actual time-frame constitutes the third factor, adding to the overall complexity of the task of finding a commonly acceptable definition. In Belgium, the law defines a shortage as an inability to dispense a particular medicine within four days (96 hours), whereas in France a shortage occurs within 72 hours only. At European level, a “time-frame” has not yet been defined, but one industry representative acknowledged that "a majority of the stakeholders would be happy to agree on a 48h time-frame."

Whether commercialisation should actually be linked to drug shortages is still a debatable point—creating a fourth challenge encountered along the way in an effort to define drug shortages. For instance, if a product is no longer marketed, should this be regarded as a bona fide medicine shortage? Some stakeholders at European level, such as the industry representatives, completely exclude product discontinuation from drug shortages, although one health authority representative said that: "A shortage should be duly acknowledged, if a particular medicine is not actually available on the market at any time after its marketing authorization has been issued."

The fifth difficulty consists in whether a measure of drug shortage severity should be considered. Several European associations address the issue of drug shortages at the level of molecules or active pharmaceutical ingredients (APIs)—a shortage is deemed to occur when a treatment alternative to a particular active substance is required. A French policy-maker said that a shortage would not occur, if an alternative treatment could be found within 72 hours, assuming a non-essential medicine is concerned. The unavailability of a product on the market will duly be defined as a shortage, although the French medicines agency (Agence Nationale de Sécurité du Médicament et des Produits de Santé, ANSM) mostly addresses shortages of essential medicines. In Belgium, a drug shortage is assessed by the Federal Agency for Medicines and Health Products (FAMHP) both in terms of the actual availability of the product itself, as well as in terms of the specific package sizes that may currently be in demand on the market (i.e. specifically required by the pharmacies).

### Dynamics and perception of drug shortages

According to the majority of interviewees, drug shortages occur daily or weekly in France and Belgium. However, their durations can vary significantly—from less than a week to an indefinitely longer period of time. One of the wholesalers from France said that 75% of the shortages lasted up to 2 weeks. Ideally, as another interviewee mentioned, one should have the shortages split up, depending on the actual causes. Shortages due to manufacturing problems generally last longer than those due to the supply chain problems. For example, a shortage due to parallel trade issues would be typically resolved within a week or two, or as soon as a new month begins. The WHO Collaborating Centre for Pharmaceutical Pricing and Reimbursement Policies defines the parallel trade in medicines within the EU as a form of arbitrage in which medicines are purchased in one Member State and then sold on to other Member States where the income levels, and hence the pricing, are usually higher [27].

According to our interviewees, when a manufacturing problem occurs, however, sometimes an entire production chain has to be taken out of commission. If products become contaminated on the production line, all batches will have to be recalled. Also, when the production site has quality problems and this comes to the attention of the regulatory institutions, the actual production run would have to be stopped and resumed only after the manufacturer has duly complied with all applicable regulatory standards, which may take up to several months.

Research participants, representing French, Belgian and European stakeholders, freely admitted in the interviews that drug shortages were being increasingly discussed, and the actual appreciation of the problem has grown within the last decade. Whether this, in fact, should actually be construed as drug shortages having increased over time was denied by some stakeholders. The interviewees representing pharmaceutical companies felt that drug shortages significantly increased with the financial crisis. A Belgian hospital pharmacist claimed that drug shortages increased significantly in comparison with the situation a decade ago, even though they are now deemed to have stabilised since last year. One French policy-maker advised caution, though: “The mediatization of drug shortages is not proportionate to their actual occurrence (…). Also, some players have a vested interest in making the problem of drug shortages more prominent, i.e. putting more pressure on price negotiations”. Using the term “mediatization” the interviewee was referring to how often the topic was raised in the media, taking into consideration that societies are shaped by and dependent on mass-media [28]. Some stakeholders pointed out that drug shortages had not caused any major public health problems as yet. Both a policy-maker and an industry representative from France readily acknowledged that drug shortages were not presently deemed a public health hazard. According to one wholesaler representative, this has caused only some inconvenience to the patients, at most.

### Determinants of drug shortages

The interviewees characterised drug shortages as a multi-layered and multi-faceted issue. They were asked to determine the actual causes of drug shortages and to reflect on their typology provided in the Birgli’s report [6]. Out of numerous reasons for the drug shortages referenced there, the most important ones are ranked further below in a descending order, based on how frequently they were marked by the interviewees:

1. manufacturing problems,
2. limited manufacturing capacity,
3. parallel distribution,
4. raw material shortages and rationing/quotas,
5. non-compliance with regulatory standards.

**Fig 1:**
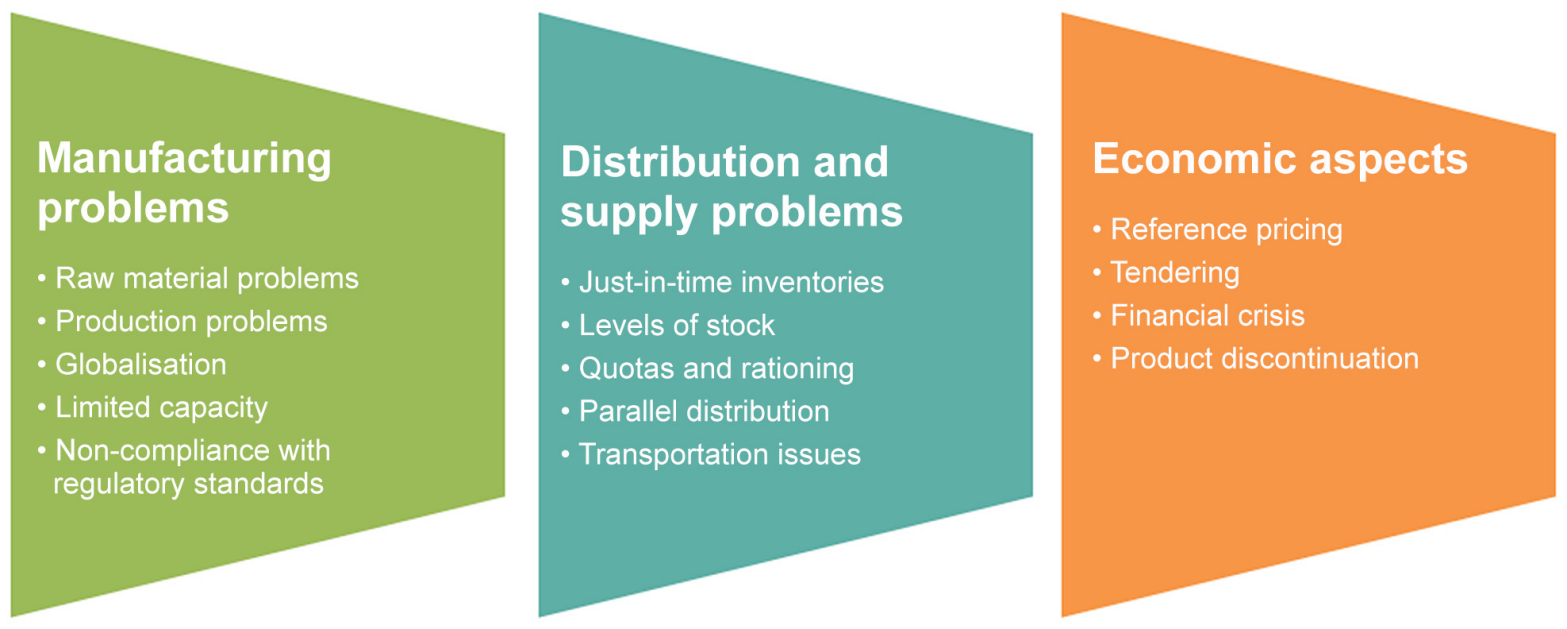
Categories and sub-categories of the reported determinants of drug shortages.

According to the interviewees, the determinants of drug shortages are split into the three main categories (Fig 1).

#### Manufacturing problems

“There has always been a potential for manufacturing problems”, said one industry representative. “We have the same quality defects as before. They do happen and we just have to get them fixed; sometimes this may lead to the closure of a factory”, explained another one. Reflecting on the typology of reasons for drug shortages, as well as utilizing their own professional experiences, the industry representatives claimed that drug shortages being the result of manufacturing problems could be linked to underlying problems with the raw materials or APIs, inadequately sized production facilities, global sourcing, production or quality issues, manufacturing capacity issues, or non-compliance with applicable regulatory standards. Several interviewees mentioned that concentration and rationalisation of pharmaceutical manufacturing actually increased the shortage hazard. A problem encountered within a single manufacturing site may well lead to major disruption on a global market. Increasingly, pharmaceutical production has been moved outside of Europe, its chain has changed over time—evolved and globalised. More countries the world over have become involved in the process. A French policy-maker said: “Presently, manufacturers source the products outside their own continent through multiple traders and, consequently, increase vulnerabilities.” Several stakeholders acknowledged that production sites of APIs had moved to China or India, which does not facilitate streamlining of their management, and therefore increases the risks of drug shortages.

Additionally, globalisation has in fact given the decision-making process an international dimension. Multinational companies might favour global economic aspects above national priorities and requirements. A hospital pharmacist volunteered an example: “Some pharmaceutical companies have a higher turn-over than Belgium’s GNP, but then the CEO of such a company might not even know where Belgium is. Commercial decisions are about business only and have nothing to do with deontology or ethics. They are just made with the shareholders in mind first and foremost.”

Also, non-compliance with applicable regulatory standards was cited as leading to shortages, following inspections on both the raw materials and the finished products. Marketing authorizations may be withdrawn due to non-conformity to the Good Manufacturing Practice (GMP) rules, according to one Belgian policy-maker. European associations, representing various pharmaceutical market stakeholders, admitted they feared the consequences of the EU Falsified Medicines Directive which introduced tougher rules to control the trade in medicines [29]. This included an authenticity feature on the outer packaging of medicines, an EU-wide logo to identify legal online pharmacies, tougher rules on the safeguards and inspection of manufacturers of active pharmaceutical ingredients, as well as stringent record-keeping requirements for wholesaler distributors. “The Directive’s impact turned out to be rather limited, though”, assured one industry representative, as the statutorily required export documents were issued in due time, in association with the key exporting countries.

#### Distribution and supply problems

Among the distribution and supply problems determining the drug shortages, the interviewees named such reasons as: just-in-time inventories and inappropriate levels of stock, parallel distribution, quotas and rationing, and transportation issues. Just-in-time inventories were acknowledged as an important reason for drug shortages. One Belgian hospital pharmacist said: “Due to prevalent economic climate, everyone started introducing just-in-time inventories. I would translate this into ‘just-too-late’ inventories. No one holds stock anymore, as stock is too expensive.” On the other hand, high levels of stock can induce drug shortages, according to some stakeholders. A hospital pharmacist offered an example: “This year we induced four stock-outs in Belgium, because we bought all the products in supply.” As the interviewees reported, when one shortage is barely resolved, another can be induced, as the dispensers of medicines tend to build up their stock disproportionately to the anticipated demand. Similarly, patients build up their medicine stockpile when a shortage has been announced, explained a French policy-maker, which further induces the shortfall. Therefore, any official communications regarding medicine shortages need to be handled with care to prevent undue confusion, pointed out the policy-maker.

Parallel distribution turned out to be an extremely controversial issue. As one representative of the European pharmacies’ environment said: “You cannot deny that parallel distribution is a factor, but you may not simply point the finger at parallel distribution, either”. In Belgium the opinions on the subject diverge. A hospital pharmacist, acting also as a policy-maker, said that parallel distribution was a major reason for drug shortages and noted that the prices of four oncological products had recently been authorized to increase up to European average values in order to prevent parallel exportation. Other policy-makers believe that parallel distribution does not affect drug shortages, as the pharmacies can still order directly from pharmaceutical companies. In France, several stakeholders referred to a critical shortage of antiretroviral medicines that occurred due to parallel exports four years earlier on, which received extensive media coverage and political involvement [30]. This case prompted significantly increased monitoring of the parallel exports of pharmaceutical products, and consequently reduced the outflow of essential medicines. Both a French policy-maker and a wholesaler’s representative claimed that parallel distribution was no longer a public health threat. Pharmaceutical companies have introduced quotas to limit the amount of products the wholesalers will receive, in an attempt to control excessive exportation. On the other hand, the wholesalers reported that quotas are difficult to handle, and therefore lead to frequent disruptions in the supply chain to the community pharmacies.

The issue of quotas is closely related to parallel trade and equally controversial amongst the stakeholders. Three different types of quotas were discussed during the interviews and identified as appearing at the following levels: (i) pharmaceutical companies, (ii) wholesaler distributors, (iii) pharmacies. Considering the first level of quotas, the number of units which a pharmaceutical company can sell on a national market can be restricted in two ways: either by national institutions through the restrictions on public expenses, or by multinational pharmaceutical companies allocating their stock to individual countries or regions, considering limited manufacturing capacity, or the actually pursued economic strategies. In this case, quotas can be based on anticipated demand, historical data, financial analyses or country-specific requirements.

The second type of quotas are those imposed by the pharmaceutical companies on particular wholesalers. The number of units a wholesaler distributor might be allocated by a manufacturer causes numerous conflicts. According to one pharmacy representative, these quotas are the primary reason for drug shortages in the community pharmacies. Usually they occur at the end of a month and last around 10 days. A French community pharmacist added: “Quotas are difficult to explain to patients (…). You have to explain that within a public health domain there are some strict economic constraints due to which the actual availability of a specific medication in demand may not be assured.” Additionally, quotas trigger conflicts between prescribers and pharmacists: “A pharmacist says he is unable to get hold of a specific medicine, even though the prescriber has received confirmation directly from the manufacturer on the product’s availability.”

Several interviewees doubted whether the quotas were well calibrated. Wholesalers urge for transparency which may then leave plenty of room for efficient planning ahead. A wholesaler representative explained quotas might well be applied and handled through different systems: “Some manufacturers will deliver more units, if a wholesaler is out of stock, but others will refuse, or deliver directly to the pharmacist. The biggest problem occurs when the manufacturers impose the ‘black-box’ quotas (…). In such cases they will not communicate with a wholesaler unless they have reached the total amount of products allocated by the manufacturers.” How quotas are actually defined is classified information, however, the stakeholders stated they were based on IMS Health data and applied mostly to highly-priced products. With regard to the third type of quotas at pharmacy level, there is a knock-on effect of the wholesaler quotas on pharmacies, as wholesalers also tend to limit the amount of products that can be ordered by a single pharmacist.

#### Economic aspects

The price component was revealed to be an important determinant of drug shortages. An industry representative indicated that registration and maintenance costs, related to e.g. pharmacovigilance and post-approval activities, have multiplied, especially after adoption of the “Pharmaceutical package”, launched in 2008 by the European Commission, containing legislative proposals aimed to form a new vision for the pharmaceutical sector (among others, it contained the proposals aiming to improve market access and accelerate research, tackle the problems with counterfeiting and illegal distribution of pharmaceuticals, facilitate patients’ access to pertinent information on the prescription-only medicines, and strengthen the pharmacovigilance systems throughout Europe).

Operating costs are much higher than ten years previously, and the industry has grown more reluctant to market medicines at any cost. Pharmaceutical industries are also undergoing restructuring as margins have dropped, reimbursement of medicines has been cut, and research and development pipelines are apparently running dry. Consequently, when a company’s profit is too small, further investments will inevitably be shelved, and this will eventually pave the way for future manufacturing problems, acknowledged a pharmaceutical company representative. He also said: “Manufacturing problems tend to increase when operations are not sustainable in general; therefore they are more a symptom than a cause.”

Furthermore, current pricing strategies have resulted in product discontinuation, especially with regard to the long-standing and the lower-priced medicines. According to a French policy-maker, some older generation antibiotics were discontinued due to low profitability. After negotiations their prices were re-evaluated, since no alternatives were available on the market, while the products were deemed essential. Another French government representative pointed out that measures to resolve shortages might well prove very expensive. Hence all the issues related to medicine availability should be taken into consideration during any price negotiations.

Purchasing strategies, including reference pricing and tendering have significantly impacted drug shortages, according to several European stakeholders. According to the definition of the WHO Collaborating Centre for Pharmaceutical Pricing and Reimbursement Policies, international or external reference pricing is the practice of using the price of a medicine in one or several countries in order to derive a benchmark or reference price for the purposes of setting or negotiating the price of the product in a particular country [27]. Tendering is a formal and competitive procurement procedure through which offers are requested, received and evaluated for the procurement of pharmaceuticals, and as a consequence of which an award is made to the tenderer (bidder) whose offer is the most advantageous [27].

“International reference pricing has affected access to medicines in the low-priced markets, because manufacturers will try to get their products out of those markets, if the product is referenced in a basket of the high-priced Member States”, said an industry representative. According to another one, tendering has two limiting aspects, i.e. adverse effects of selecting a sole supplier, and having a tender awarded to a supplier exclusively on pricing considerations, whereas the product availability aspect also needs to be taken on board. There is always a risk that an exclusive manufacturer might not be able to cope with an unexpected surge in demand for the products. Besides, other manufacturers will cease production and will therefore be unable to compensate for the shortage, should the exclusive manufacturer who won the tender, run into serious production problems. A French policy-maker concurred: “Often the cheapest deal is made rather than the best one. Eventually, if a shortage occurs, you end up paying, anyway.”

### Role of the European and national institutions

The role of the European Union was referred to by the interviewed stakeholders as related primarily to overall coordination. According to one wholesaler representative, European institutions should monitor manufacturing problems, ensure transparency, and give an early warning with regard to any centrally registered drug shortages. However, this interviewee did not suggest any specific institution. Representatives of the European associations put forward the need for a steering committee at the European level to take the lead.

According to a representative of a medicine agency, working groups at the European Medicines Agency (EMA) have been set up to hammer out a European strategy addressing imminent and presently occurring drug shortages. Currently, the Member States are individually setting up rules and regulations to protect their own markets and patients, thereby creating additional imbalances, declared a French policy-maker. For some essential products, an EU approach should be considered, to assure availability across the entire Union. Currently, when a shortage occurs, Member States are in direct competition with each other instead of working hand in hand to resolve the issue effectively, he added.

A community pharmacy representative was surprised by the lack of interest concerning drug shortage issues at the European level, which (as he claimed) was the result of holding back from various conflicts and tensions. In his view the European Commission would not like to intervene in the pricing and reimbursement policies, even though it believed it was actually the principal reason for the shortages. Consequently, medicines are moved from the markets where the prices are lower to the ones where they can fetch higher prices. “Parallel distribution is a pure creation of the European internal market. (…) So I suppose you could say, to the extent that parallel distribution is an issue, that it is an European issue. ‘European laws’ allow you to set quotas for public health reasons, but nobody knows what the correct position is, or where to actually draw the line”, continued the interviewee.

A wholesaler representative admitted that there is no specific guidance on how the Member States should deal with Article 81, Directive 2001/83/EC, and the principles of free movement of goods, and the competition law. It is difficult for the Commission to officially communicate on the topic. However, within the very fold of the Commission itself quite divergent opinions are expressed about the role of parallel trade in the pharmaceutical industry, said an interviewee.

One industry representative added: “You might find DG SANCO and DG Enterprise torn on how to address the issue. Member States will not want them to set forth any guidelines on anything because it is their sovereign right.” The DG SANCO and DG Enterprise are abbreviated names of two departments of the European Commission. The first one deals with public health issues and as of 2015 it has been named DG Sante (the Directorate-General for Health and Food Safety). The other one deals with industrial policies and as of 2015 it has been named DG Growth (the Directorate-General for Internal Market, Industry, Entrepreneurship and Small and Medium-Sized Enterprises).

On a national level, Member States have arranged for early warning systems. One Belgian pharmacist believed that respective national agencies should primarily be responsible for collecting information on any drug shortages (including information on alternative treatments) in real time, and then make it freely available to all healthcare providers. Second, the government should deal with quality and production problems. Another Belgian pharmacist argued: “The government becomes aware of the shortages far too late, because companies do not wish to communicate with anyone about the shortages. Companies do not want to be registered on the shortage list to prevent their competitors from submitting a request for the derogation of imports.” In France, a wholesaler believed the government should also raise awareness among the stakeholders to stimulate collaboration and introduce specific measures against drug shortages. European representatives of stakeholders’ associations pointed out the influence of national payers and respective ministries of health on pricing and reimbursement issues.

## Discussion

This study highlights several important issues underlying drug shortages in the European Union, thus aiding understanding of a rather complex nature of this problem, its origins and potential resolution methods. Drug shortages are a complex phenomenon of global impact [5]. Within Europe, drug shortages have been investigated so far only within a limited scope [1, 31–33].

As our interviewees reported, the differences in the terminology applied to describe the drug shortages are encountered in Belgium and France. Admittedly, a diversity of definitions are also internationally acknowledged. The International Society for Pharmaceutical Engineering (ISPE) defines them as “a situation in which the total supply of an approved (by an appropriate Health Authority) medicine is inadequate to meet the current projections or projected demand at the user level” [34].

The Food and Drug Administration (FDA) defines a drug shortage more narrowly, as “a situation in which the total supply of all clinically interchangeable versions of an FDA-regulated medicine is inadequate to meet the current or projected demand at the user level”. Additionally, the FDA adds a public health factor to it, focusing on the shortages of medically necessary products that have a significant effect on the population’s health [35]. The definition used by the FDA is based on section 506C(h)(2) of the Food, Drug and Cosmetic Act, according to which a drug shortage is “a period of time when the demand or projected demand for the drug within the United States exceeds the supply of the drug” [36]. On the other hand, the American Society of Health-System Pharmacists (ASHP) defines a drug shortage as “a supply issue that affects how the pharmacy prepares or dispenses a medicine product or influences patient care when prescribers must use an alternative agent”. In those cases, a broader definition is used in which a drug shortage has the potential to compromise patient care [37].

Similar to the FDA, the European Federation of Pharmaceutical Industries and Associations (EFPIA) defines medicine shortages as “a crisis situation caused by the inability of any Market Authorization Holder (MAH) to supply a medicine with a specific API to a market over an extended period of time resulting in the unavailability of this medication for patients” [38]. Finally, the EMA takes into account only drug shortages which could lead to public health crises that arise due to disruptions within the manufacturing process, caused by manufacturing or GMP compliance problems [39].

The difficulty in defining and categorizing drug shortages may to some extent be related to the lack of transparent quantitative data on drug shortages [6,30]. As the material from the interviews with stakeholders from various specializations demonstrates, they still struggle to agree on a common terminology. The term “shortage” does not seem broad enough to describe the different aspects of a shortage and creates confusion amongst the stakeholders. Drug shortages may be perceived from different angles with each of the stakeholders adhering to his own view. A shortage at the manufacturer’s level is not due to the same causes and consequences as a shortage at the pharmacist’s level. Nonetheless, the perspective of a manufacturer and a pharmacist is adopted, respectively, to describe a shortage in line with applicable constraints of Belgian and French legislation. It should also be clearly said at this juncture, however, that in both Belgian and French legislation instead of making consistent use of the term “shortage”, the terms “unavailability of a medicine” and “disruption of supply” are used interchangeably.

As may readily be seen from our interviews, defining and categorizing drug shortages is also difficult, due to the fact that one has to decide on the actual time-frame, whether to include a measure of severity, and whether to include any discontinued products. Consequently, making use of a single word may not be entirely appropriate, as it would most obviously fail to embrace the different aspects of drug shortages. Depending on the cause and the severity of a shortage, different terminology should be applied instead. As the issue of medicine shortages is discussed in more depth, researchers, analysts and readers of thematic reports should become fully aware of the inconsistency within the currently applied nomenclature, with the need for a comprehensive glossary of pertinent terms becoming paramount.

Although the stakeholders interviewed for the purpose of this study do agree that drug shortages occur regularly, it is difficult to assess the actual severity of the situation. In Europe, the exact figures regarding drug shortages are not available, despite an increasing number of reports and studies on the issue [6,8,31,32,38,40–43]. The EMA has recently compiled a shortage catalogue which comprises information on drug shortages that either affect, or are likely to affect more than one of the European Member States [44]. However, the shortage catalogue fails to provide a comprehensive listing of drug shortages, as most of them are dealt with at a national level.

In the winter of 2012–2013, the European Association of Hospital Pharmacists (EAHP) carried out a survey in hospital pharmacies [43]. The study revealed that 99% of hospital pharmacies experienced drug shortages in the previous year, with 63% of the participants reporting weekly, or sometimes daily, shortages. Additionally, 77% of hospital pharmacies reported that drug shortages became worse in the previous year. Generic and original medicines seemed equally affected, but differed when the respondent’s country was taken into account. The areas of medicine that were most affected included oncology (70.6%) and cardiovascular disorders (43.8%).

As our interviewees admitted, both hospital and community pharmacies were also affected by drug shortages. This seems to corroborate the findings published elsewhere. In late 2012, the Pharmaceutical Group of the European Union (PGEU) conducted a survey among its members [7,45]. All respondents reported drug shortages, although their severity varied. According to the survey, the situation grew worse in the past 12 months, and a broad range of medicines was affected, including generic and branded medicines. As the results yielded by the present study have partly corroborated, some pharmacy associations regularly produce a list of medicines in short supply (e.g. the Irish Pharmacy Union and the Dutch Royal Pharmacy Society) [45]. Likewise, in other countries the regulators have set up specific websites in order to keep the stakeholders regularly advised about any unavailability of medicines, whereupon pertinent information is mostly provided by the manufacturers on a voluntary basis (e.g. Belgium, but also Italy and Lithuania) [45].

As far as Belgium is concerned, 143 products were listed by the FAMHP as temporarily unavailable (a counting unit including product name, formulation and dose) in March 2014 [46]. Additionally, 11 medicines were separately reported, as their unavailability could lead to a public health hazard or pose a tangible threat to patients who should receive the treatment. Noticeably, the two major therapy areas in which shortages occurred predominantly in Belgium, were the disorders of the nervous system and of the cardiovascular system. The majority of the reported drug shortages pertained to the generic products.

With regard to France, the ANSM reported 34 drug shortages, 13 product discontinuations, 13 products with an imminent shortage, and 20 products with restored availability (a counting unit including the product name and its formulation) in March 2014 [47]. The therapy area in which most of the drug shortages occurred concerned treatments targeting diseases of the nervous system. This was followed by the shortages of anti-infective, anti-neoplastic and immunomodulatory medicines.

**Fig 2:**
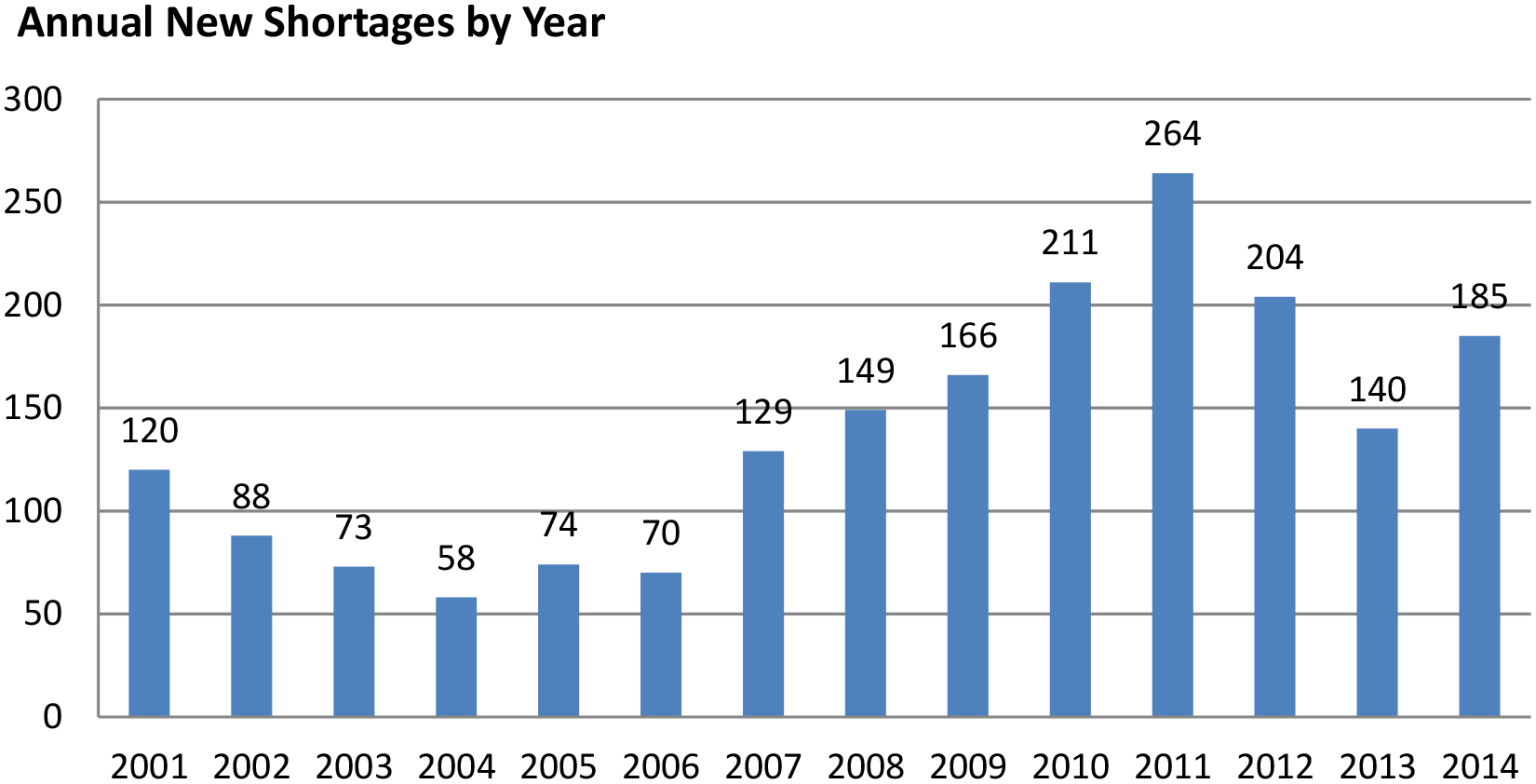
Drug shortages in the USA spanning January 2001—December 2014 [58].

It seems that drug shortage issues are more prominent in the USA than in the EU, as an increased number of shortages has been reported there within the past decades, intensifying even more in recent years [48,49]. Within the period spanning 2006–2011, the ASHP reported a three-fold increase in drug shortages [49–51]. It is also shown in Fig 2, where the adjacent columns represent the number of new shortages within each year. On 8 January 2014, there were 239 drug shortages published on the ASHP website [52]. The FDA registered by generic name or active ingredient 73 medicines currently in shortage and 33 with the status of resolved shortages (as of 15 February 2015) [53]. The drug shortage problem mostly affects older generic injectable medicines in the therapeutic domains of oncology and anti-infective treatments [54].

Whenever the actual determinants of drug shortages were concerned, no single aspect had been decisively indicated by our interviewees as dominant. However, the economic aspects seemed to have claimed a leading role, effectively determining commercial decisions of the pharmaceutical market stakeholders. Manufacturing problems and product supply issues were suggested in other published sources as the most important reasons for shortages [31,54]. Similar to the situation in the EU, the multi-causal character of drug shortages was also reported in the USA [51].

Rationalisation and consolidation of the industry has increased vulnerabilities in the manufacturing and the supply chain. Nowadays, there are fewer manufacturing sites and APIs are sourced outside Europe. Furthermore, manufacturers use the same production line for multiple products, which can limit production capacity [55]. Consequently, a specific problem encountered within one production site may well lead to a global crisis [51,55]. In general, manufacturing problems also tend to increase when operations are not sustainable. Besides, economic aspects are inter-related with distribution and supply problems. In order to reduce the cost of inventories and optimise cash flows, the stakeholders tend to opt for the just-in-time inventory management, which increases overall susceptibility to shortages when a change in demand or supply should unexpectedly occur.

Parallel distribution is also based on economic decisions, as the products are exported to the higher-priced countries and subsequently sold there for extra profit. Pharmaceutical companies are the main exporters of pharmaceutical products in France [30]. However, their anxiety with regard to parallel export that might potentially be pursued by the wholesale distributors, or their belief that this might already be going on, inevitably lead the pharmaceutical companies to introduce quotas as an effective counter-measure. Quotas are also implemented by (multinational) pharmaceutical companies that allocate stock to individual countries in view of a limited manufacturing capacity, or specifically tailored economic strategies. Whether it is actually the quotas or parallel trade that leads directly to drug shortages, is a hotly debated issue amongst the stakeholders, according to our study participants. The consequences may be quite significant, however, and affect mostly the medicines dispensed by the community pharmacies.

Drug shortages may also be impacted directly by some economic aspects, according to our study, as well as to other published sources. For example, a lack of market attractiveness and low profitability may prompt the discontinuation of some long-standing, or lower-priced products, e.g. antibiotics and oncologic medicines [1,5,10,48]. Pricing strategies, such as reference pricing and tendering, may actually influence a manufacturer’s decision to keep a product on the market. A company might decide to pull out of production, if a tender is lost, or remove its product from the market, should it lead to a loss of profits in a higher-priced country [6]. Finally, regulatory compliance requirements related to pharmacovigilance and post-authorisation activities may also prove costly [6,39].

Manufacturing problems and quality defects seem to influence the injectables more often than other formulations, due to the complexity of their production [31,56]. The economic determinants seem to exert a particularly strong influence on the shortages of generic injectables, at least in the USA [31,56]. In one of the recently published studies the generics were not reported as outnumbering the branded medicines within the statistics on drug shortages in Belgium, the Netherlands, the United Kingdom, Germany, Italy, Spain and France [31]. However, according to its authors, this could actually be attributable to the inherent limitations of the reporting systems in those countries. When essential medicines and oncology drugs were studied (sub-analysis of the same study results), overall share of generic injectables in the shortages increased appreciably. Similarly, in the USA, the majority of pharmaceuticals in short supply had oncology indications [31,51,54,57].

The present study corroborates the global character of drug shortages, as well as the importance of international links and relations in understanding and coping with this problem. In spite of that, the participants’ statements indicate that there seems to be a profound lack of interest in this issue at the European level.

On the one hand, respective national institutions keep on developing and implementing diverse regulations regarding prices and general availability of medicines. In fact, national policies do play a pivotal role, as they can effectively influence commercial decisions and, consequently, overall sustainability of the market. As pricing negotiations may well persuade a manufacturer to keep a specific product on the market, the institutions in charge of regulating the prices of medicines should closely collaborate with the ones regulating the actual availability of the medicines. National authorities need to put some pressure on the stakeholders in order to find a middle ground between their goals and objectives and the actual implementation of specific measures to ensure a continuous supply of medicines, with a view to effectively protecting public health. In turn, policy-makers are challenged to reduce health care expenditure in the services without compromising the quality and availability of the products. Eventually, the responsibility for access to and the supply of medicines rests with the respective national authorities.

On the other hand, as our interviews seem to imply, the rules and regulations which are individually set up by the national authorities, primarily in order to protect their market and patients, create imbalances at the European level. The effect of the interaction between Member States on drug shortages therefore needs to be monitored at the European level. The European institutions need to coordinate the common European approach to assure the availability of medicines, as well as to deal effectively with the manufacturing problems impacting the EU as a whole. Also the effect of European directives and regulations should be monitored by the EU institutions. Non-compliance with applicable regulatory standards may lead to drug shortages, e.g. due to non-conformity with the GMP rules, or (at least potentially) as a consequence of duly implementing the provisions of the EU Falsified Medicines Directive. Based on our interviews, the Member States should also be specifically guided on how to deal with applicable European directives and regulations.

The present study has several limitations, partly inherent to the adopted methodology and a rather complex nature of the problem. They pertain both to the country selection and the population sample of the interviewed stakeholders. Although the issue of drug shortages is of paramount importance across several countries, only two of them were assessed. Some important stakeholder groups were not included due to the actual study design and specific time-line constraints. For instance, the representatives of patients and prescribers might well have revealed other important aspects of drug shortages. However, the in-person, semi-structured interviews with 21 professionals active in relevant institutions and organisations facilitated an in-depth and broad appraisal of the problem under study. The high response rate gave us the opportunity to assess the different perspectives and complexities which characterise all drug shortages.

To the best of our knowledge, this is the first qualitative study to investigate the characteristics, the determinants, and the problem drivers of drug shortages, focusing on this particular group of countries, while also adopting the European Union’s perspective. Additional studies should obviously be pursued to better quantify and further identify the underlying causes of drug shortages in the entire EU, in order to develop long-term prevention strategies and strengthen any specifically focused mitigation responses.

## Conclusions

Drug shortages have increasingly been reported in Belgium and France. They occur daily or weekly, and their durations can vary significantly—from less than a week to an indefinitely longer period of time. Appreciation of this issue has increased within the last decade. However, whether this should actually be construed as the drug shortages having, in fact, increased over time was denied by some stakeholders.

Defining drug shortages was found to be exceedingly complicated by a great diversity of opinions on the level at which all drug shortages should be assessed, the appropriate time-frame applied, the issue of linking commercialisation to shortages, and finally—by the severity of the problem itself. The commonly agreed terminology should effectively be developed to facilitate popular appreciation of its underlying causes, intensity and complex character.

Three groups of determinants of drug shortages were identified throughout the present study: manufacturing problems, distribution and supply problems, and problems related to some economic aspects. Manufacturing problems stem from concentration and rationalisation of pharmaceutical manufacturing, as well as globalisation. Keeping communication lines with the stakeholders open at all times was found to be crucial in order to minimise the distribution and supply problems. Both parallel distribution and quotas turned out to be extremely controversial issues. Economic aspects seem to play a pivotal role in drug shortages, effectively impacting commercial decisions made by all of the interviewed stakeholders. Rationalisation and consolidation of the industry has increased vulnerabilities in the production and supply chain. Furthermore, pricing and reimbursement strategies seem to have a considerable impact on drug shortages.

EU institutions need to coordinate legal and organisational strategies to address the issue of drug shortages between all Member States. Presently, they seem to express rather low, if any, interest in having this problem effectively resolved. Far more focused and dedicated collaboration among the Member States might well prove instrumental in coping with drug shortages throughout the European Union more effectively.

## Supporting Information

S1 Text Interview guide—set of questions for manufacturers. (DOCX)

S2 Text Interview guide—set of questions for wholesalers. (DOCX)

S3 Text Interview guide—set of questions for pharmacists. (DOCX)

S4 Text Interview guide—set of questions for policy-makers. (DOCX)
