# Supplementary material for: A Qualitative Approach to a Better Understanding of the Problems Underlying Drug Shortages, as Viewed from Belgian, French and the European Union’s Perspectives
Source: PLoS One. 2015 May 5;10(5):e0125691. doi: 10.1371/journal.pone.0125691 (PMC4420462; doi:10.1371/journal.pone.0125691)
Supplement: S1 Text — (DOCX) [file pone.0125691.s001.docx]

**supplementary material**

**Text S1. Interview guide – manufacturers**

**General questions**

How do you understand the term “drug shortages”?

If the answer is rather general, ask more specifically what they consider as, e.g.:

- Unavailability due to a shortage at the level of wholesaler or pharmaceutical company.
- Unavailability due to an inadequate amount of stock at the level of a pharmacy (a trade-off between the cost of stock and a probability of having a drug shortage).
- In this context we will define drug shortages as a deficiency in the supply of a medicinal product which hinders meeting the demand of the product at a patient level.
- Do we count in the unavailability the drugs whose production has been discontinued in the country by a pharmaceutical company, the drugs that have never been registered in the country, and the new drugs pending registration by a pertinent authority?

- How often have you been encountering the problem of drug shortages in your own professional

practice?

Please tick off the appropriate box, if applicable.

**Table 1**. Frequency versus duration of drug shortages.

| Duration | Frequency | | | | |
| --- | --- | --- | --- | --- | --- |
|  | Every week | Every month | Every 3 months | Every 6 months | Every year |
| Less than 1 week |  |  |  |  |  |
| 1 week to 1 month |  |  |  |  |  |
| More than 1 month |  |  |  |  |  |
| Undefined or unlimited |  |  |  |  |  |

What are the dynamics of drug shortages in recent years? Has the problem intensified or decreased?

What are the reasons for drug shortages in your opinion?

Ask what they regard as the principal cause

- - Technical problem in manufacturing or distribution processes?
  - Increasing demand, limited production capacity?
  - Shortage of raw materials?
  - Other...

Show Table 2 and ask questions about relevant reasons for the shortages: according to some researchers there are predictable and unpredictable reasons for drug shortages. In your opinion what is the influence of each of those factors? Do you think other reasons could be added to this table? Could you rate their importance (1-3)?

**Table 2.** Reasons for drug shortages *

| **Nr Unpredictable** | **Nr. Predictable** |
| --- | --- |
| - Natural disasters | - Product discontinuation |
| - Manufacturing problems | - Industry consolidation (M&A) |
| - Raw material shortages | - Limited manufacturing capacity |
| - Non-compliance with regulatory standards | - Just-in-time inventories |
| - Packaging shortages | - Rationing / quotas |
| - Unexpected demand | - Deliberately induced shortages to manipulate the pricing |
| - Epidemics | - Market shifts |
| - Parallel distribution - Competitive issues | - Launch of a new competitor, new formulation, or patent expiry - Other ………………………………………………. |
| - Foreign currency exchange effect |  |
| - Sovereign issues (financial crisis, debt, default) - Other ……………………………………………. |  |

* (The table is based on: Birgli® ag (2013) An Evaluation of Medicines Shortages in Europe with a more in-depth review of these in France, Greece, Poland, Spain, and the United Kingdom. Zug. Available: http://static.correofarmaceutico.com/docs/2013/10/21/evaluation.pdf. Accessed 8 January 2014.)

What are the most important consequences of drug shortages in your opinion ?

**Legal and organizational aspects**

Do you know about any laws and/or regulations that may influence the occurrence of drug shortages?

Provide a list and indicate the ones most important to your institution with regard to the drug shortages?

Could the realization of the GMP rules influence, in any way, the occurrence of drug shortages?

Could the EU regulations on falsified medicines influence in any way the occurrence of drug shortages ?

Do you think that new legal regulations and organizational solutions could be implemented in order to reduce the problem of drug shortages? Please, address this issue in more detail.

Do you know any examples of such regulations in place in other countries?

Which ones out of the existing legal regulations and organizational solutions could be changed in order to reduce the problem of drug shortages ?

What role do national and EU institutions play in drug shortages ? Do you think that national and EU institutions provide enough support to prevent drug shortages? If not, what other measures could be put in place?

Do the quotas imposed by country or by institutions influence in any way the drug shortages? How are they implemented and who sets them?

What is the influence of parallel trade on the drug shortages?

**Pricing and reimbursement**

How could pricing and reimbursement policies influence the drug shortages?

Do you think that the pressure put on drug pricing in your country can have an influence on the quotas?

Do you think that the pressure put on drug pricing in your country is higher than in other EU countries?

Does the economic slowdown influence the pharmaceutical market and could it be one of the underlying reasons for the drug shortages?

**Communication**

What do you think about the communication regarding the drug shortages?

Who should be responsible for the communication regarding the drug shortages?

When should drug shortages be announced?

How should drug shortages be announced?

Does the manufacturer warn customers about a prospective or an existing drug shortage? How does that happen and when exactly is the customer warned?

**Solutions**

Which specific measures are taken by your institution to reduce or prevent the drug shortages? Are there any other measures, in your view, that could be implemented?

How could other stakeholders in the supply chain contribute to reducing the drug shortages?

In some countries, special drug shortage committees have been set up to reduce the drug shortages. Do you think such an institution might be useful in a particular country?
