# Supplementary material for: A Qualitative Approach to a Better Understanding of the Problems Underlying Drug Shortages, as Viewed from Belgian, French and the European Union’s Perspectives
Source: PLoS One. 2015 May 5;10(5):e0125691. doi: 10.1371/journal.pone.0125691 (PMC4420462; doi:10.1371/journal.pone.0125691)
Supplement: S2 Text — (DOCX) [file pone.0125691.s002.docx]

**supplementary material**

**Text S2. Interview guide – wholesalers**

**General questions**

How do you understand the term “drug shortages”?

If the answer is rather general, ask more specifically what they consider as e.g.:

- Unavailability due to shortage at the level of wholesaler or pharmaceutical company
- Unavailability due to an inadequate amount of stock at the level of the pharmacy (a trade-off between the cost of stock and a probability of having a drug shortage).
- In this context we will define drug shortages as a deficiency in the supply of a medicinal product which hinders meeting the demand of the product at a patient level.
- Do we count in the unavailability the drugs whose production has been discontinued in the country by a pharmaceutical company, the drugs that have never been registered in the country, and the new drugs pending registration by a pertinent authority?
- How often have you been encountering the problem of drug shortages in your own professional practice?

Please tick off the appropriate box, if applicable.

**Table 1**. Frequency versus duration of drug shortages

| Duration | Frequency | | | | |
| --- | --- | --- | --- | --- | --- |
|  | Every week | Every month | Every 3 months | Every 6 months | Every year |
| Less than 1 week |  |  |  |  |  |
| 1 week to 1 month |  |  |  |  |  |
| More than 1 month |  |  |  |  |  |
| Undefined or unlimited |  |  |  |  |  |

What are the dynamics of drug shortage in recent years? Has the problem intensified or decreased?

How do drug shortages manifest themselves and influence your institution?

What are the reasons for drug shortages in your opinion? Ask what they regard as the principal causes

then show them the list.

Show Table 2 and ask questions about relevant reasons for the shortages: According to some researchers there are predictable and unpredictable reasons for drug shortages. In your opinion what is the influence of each of those factors? Do you think other reasons could be added to this table? Could you rate their importance (1-3)?

**Table 2.** Reasons for drug shortages *

| **Nr Unpredictable** | **Nr. Predictable** |
| --- | --- |
| - Natural Disasters | - Product discontinuation |
| - Manufacturing problems | - Industry consolidation (M&A) |
| - Raw Material Shortages | - Limited manufacturing capacity |
| - Non-compliance with regulatory standards | - Just-in-time inventories |
| - Packaging shortages | - Rationing / quotas |
| - Unexpected demand | - Deliberately induced shortages to manipulate the pricing |
| - Epidemics | - Market shifts |
| - Parallel distribution - Competitive issues | - Launch of a new competitor, new formulation, or patent expiry - Other ………………………………………………. |
| - Foreign currency exchange effect |  |
| - Sovereign issues (financial crisis, debt, default) - Other ……………………………………………. |  |

* (The table is based on: Birgli® ag (2013) An Evaluation of Medicines Shortages in Europe with a more in-depth review of these in France, Greece, Poland, Spain, and the United Kingdom. Zug. Available: http://static.correofarmaceutico.com/docs/2013/10/21/evaluation.pdf. Accessed 8 January 2014.)

What are the most important consequences of drug shortages in your opinion ?

**Legal and organizational aspects**

Do you know about any laws and/or regulations that may influence the occurrence of drug shortages?

Provide a list and indicate the ones most important to your institution with regard to the drug shortages?

Do you think new legal regulations and organizational solutions could be implemented in order to reduce the problem of drug shortages? Please, address this issue in more detail.

Do you know any examples of such regulations in place in other countries?

Which ones out of the existing legal regulations and organizational solutions could be changed in order to reduce the problem of drug shortages ?

What role do national and EU institutions play in drug shortages ? Do you think that national and EU institutions provide enough support to prevent drug shortages? If not, what other measures could be put in place?

Could the rules of stock management implemented in your organization somehow influence the occurrence of the drug shortages?

Opinion of the wholesalers about the legal requirements on stock levels?

What is your opinion on the regulation stipulating that 66% of marketed products should be covered by the stock held by the wholesalers? Do you think this is too much or not enough?

What is your opinion on the regulation stipulating that 1 month worth of mean annual consumption should be available in the stock held by the wholesaler? Do you think this is too much or not enough?

What do you think is the influence of the supply quotas on the drug shortages?

What is the influence of parallel trade on the drug shortages?

What do you think is the influence of pricing and reimbursement policy on the drug shortages?

**Communication**

What do you think about the communication regarding the drug shortages?

Who should be responsible for the communication regarding the drug shortages?

When should drug shortages be announced?

How should drug shortages be announced?

Are the wholesalers warned of prospective drug shortages before they occur? If yes, who provides the information and how is this information disseminated?

Do wholesalers warn customers of a prospective or an existing shortage? If yes, which method is used to communicate this information?

**Solutions**

Which specific action do the wholesalers take when a drug at issue is struck off the ordering list they use?

Which specific measures are taken by your institution to reduce or prevent the drug shortages? Are there any other measures, in your view, that could be implemented?

Do the wholesalers suggest any transitory solutions to alleviate the problem?

How could other stakeholders in the supply chain contribute to reducing the drug shortages?

In some countries, special drug shortage committees have been set up to reduce the drug shortages. Do you think such an institution might be useful in a particular country?
