# Supplementary material for: A Qualitative Approach to a Better Understanding of the Problems Underlying Drug Shortages, as Viewed from Belgian, French and the European Union’s Perspectives
Source: PLoS One. 2015 May 5;10(5):e0125691. doi: 10.1371/journal.pone.0125691 (PMC4420462; doi:10.1371/journal.pone.0125691)
Supplement: S4 Text — (DOCX) [file pone.0125691.s004.docx]

**supplementary material**

**Text S4. Interview guide – policy-makers**

**General questions**

How do you understand the term “drug shortages”?

If the answer is rather general ask more specifically what they consider as e.g.:

- Unavailability due to shortage at the level of wholesaler or pharmaceutical company
- Unavailability due to an inadequate amount of stock at the level of the pharmacy (a trade-off between the cost of stock and a probability of having a drug shortage).
- In this context we will define drug shortages as a deficiency in the supply of a medicinal product which hinders meeting the demand of the product at a patient level.
- Do we count in the unavailability the drugs whose production has been discontinued in the country by a pharmaceutical company, the drugs that have never been registered in the country, and the new drugs pending registration by a pertinent authority?
- How often have you been encountering problems of drug shortages in your own professional practice?

Please tick off the appropriate box, if applicable.

**Table 1**. Frequency versus duration of drug shortages

| Duration | Frequency | | | | |
| --- | --- | --- | --- | --- | --- |
|  | Every week | Every month | Every 3 months | Every 6 months | Every year |
| Less than 1 week |  |  |  |  |  |
| 1 week to 1 month |  |  |  |  |  |
| More than 1 month |  |  |  |  |  |
| Undefined or unlimited |  |  |  |  |  |

What are the dynamics of drug shortage in recent years? Has the problem intensified or decreased?

What are the reasons for the drug shortages in your opinion?

Ask what they regard as the principal cause and then show the list.

Show Table 2 and ask questions about relevant reasons for shortages: According to some researchers there are predictable and unpredictable reasons for drug shortages. In your opinion what are the influence of each of those factors? Do you think other reasons could be added to this table? Could you rate their importance (1-3)?

**Table 2.** Reasons for drug shortages *

| **Nr Unpredictable** | **Nr. Predictable** |
| --- | --- |
| - Natural disasters | - Product discontinuation |
| - Manufacturing problems | - Industry consolidation (M&A) |
| - Raw material shortages | - Limited manufacturing capacity |
| - Non-compliance with regulatory standards | - Just-in-time inventories |
| - Packaging shortages | - Rationing / quotas |
| - Unexpected demand | - Deliberately induced shortages to manipulate the pricing |
| - Epidemics | - Market shifts |
| - Parallel distribution - Competitive issues | - Launch of a new competitor, new formulation, or patent expiry - Other ………………………………………………. |
| - Foreign currency exchange effect |  |
| - Sovereign issues (financial crisis, debt, default) - Other ……………………………………………. |  |

* (The table is based on: Birgli® ag (2013) An Evaluation of Medicines Shortages in Europe with a more in-depth review of these in France, Greece, Poland, Spain, and the United Kingdom. Zug. Available: http://static.correofarmaceutico.com/docs/2013/10/21/evaluation.pdf. Accessed 8 January 2014.)

What are the most important consequences of drug shortages in your opinion?

**Legal and organizational aspects**

Do you know about any laws and/or regulations that may influence the occurrence of drug shortages?

Provide a list and indicate the ones most important to your institution with regard to the drug shortages?

What role do national and EU institutions play in drug shortages ? Do you think that EU institutions provide enough support to prevent drug shortages? If not, what other measures could be put in place?

Please offer your opinion on the legal requirements on the stock levels that should be maintained by the

wholesalers?

What is your opinion on the regulation stipulating that 66% of marketed products should be covered by the stock held by the wholesalers? Do you think this is too much or not enough?

What is your opinion on the regulation that 1 month of mean annual consumption should be available in stock by the wholesaler? Do you think this is too much or not enough?

What is your opinion on the regulation stipulating that 1 month worth of mean annual consumption should be available in the stock held by the wholesaler? Do you think this is too much or not enough?

In which way could the participants in the supply chain exert any influence on the drug shortages?

What do you think is the influence of the supply quotas on the drug shortages?

Are the nationally imposed quotas or the ones imposed by institutions that actually impact overall drug availability on the market? How are they implemented and who actually sets them?

Can globalization influence the problem of drug shortages? If yes, in what way?

What is the influence of parallel trade on the drug shortages?

Which specific measures are being taken by the health authorities to reduce drug shortages? Are there any other measures that should be implemented?

**Pricing and reimbursement**

How could pharmaceutical pricing and reimbursement policies influence problems of the drug shortages?

Do you think that the pressure put on drug pricing in your country can have an influence on the quotas?

Do you think that the pressure put on drug pricing in your country is higher than in other EU countries?

Do you think the newly introduced legal regulations and organizational solutions should be implemented in order to reduce the problem of drug shortages? Please, address them in more detail. Do you know any specific examples of such regulations in other countries?

Does the economic slowdown influence the pharmaceutical market and could it be one of the underlying reasons for the drug shortages?

**Communication**

What do you think about the communication regarding the drug shortages?

Are the health authorities warned of prospective drug shortages before they occur?

Who should be responsible for the communication regarding the drug shortages?

When should drug shortages be announced?

How should drug shortages be announced?

Do you think the stakeholders are aware of how drug shortages should be reported?

Do the health authorities warn customers of a prospective or an existing shortage? How is this done and when is the customer actually warned?

**Solutions**

Do the health authorities suggest any transitory solutions to alleviate the problem? Are there any other measures, in your view, that could be implemented?

How could other stakeholders in the supply chain contribute to reducing the drug shortages?

In some countries, special drug shortage committees have been set up to reduce the drug shortages. Do you think such an institution might be useful in a particular country?
